# Supplementary material for: Coordination of shoot apical meristem shape and identity by APETALA2 during floral transition in Arabidopsis
Source: Nat Commun. 2024 Aug 13;15:6930. doi: 10.1038/s41467-024-51341-6 (PMC11322546; doi:10.1038/s41467-024-51341-6)
Supplement: Supplementary file 1 — Supplementary Information [file 41467_2024_51341_MOESM1_ESM.pdf]

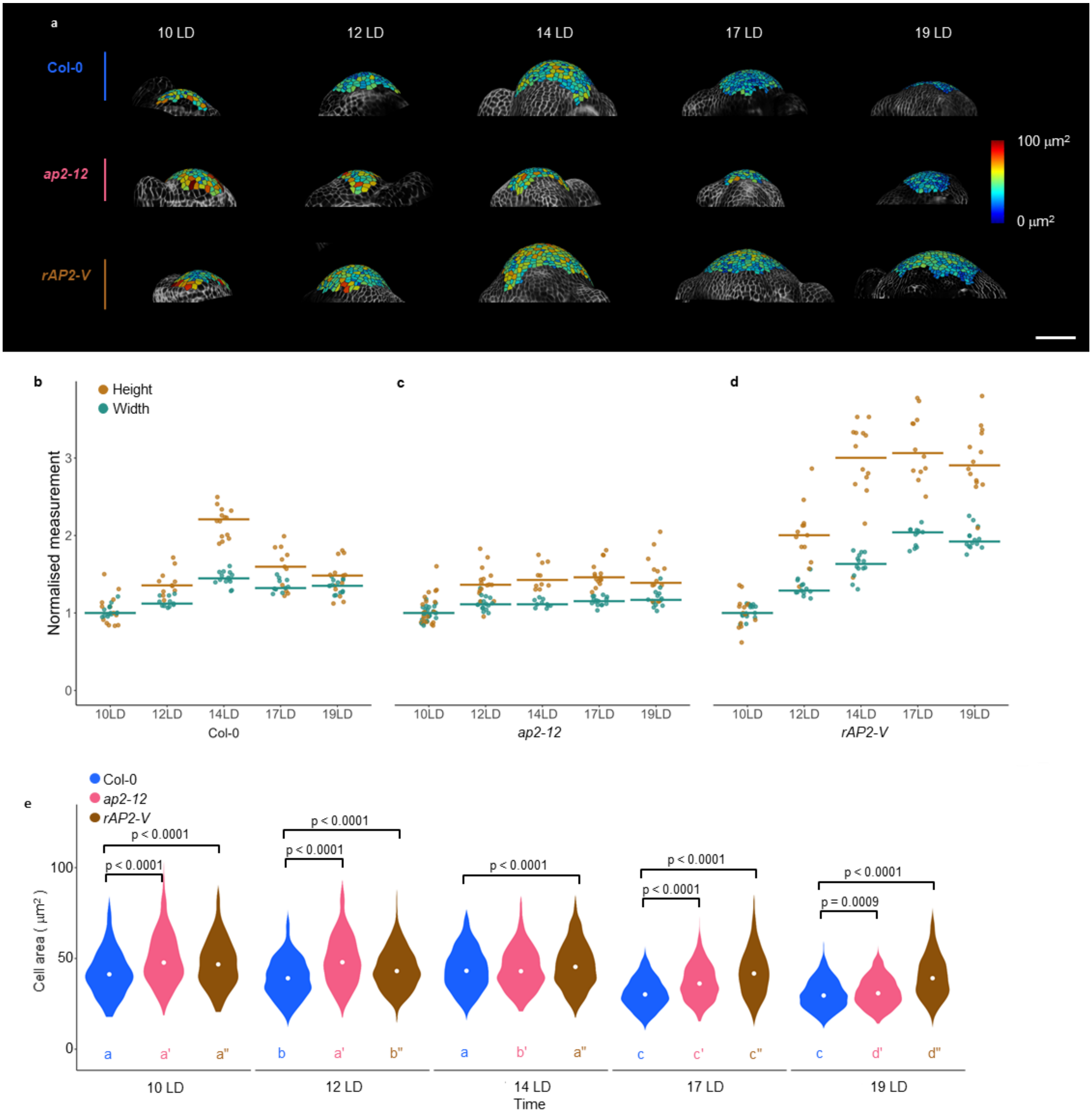

Supplementary Figure 1. Supplementary data for Fig. 1: Additional characterization of meristem morphology under continuous long day conditions. (a) Lateral view of the heatmap quantification of cell area in the meristem region of Col-0, *ap2-12* and *rAP2-VENUS* (*rAP2-V*) SAMs grown under continuous long days (LDs) shown in Fig. 1d. White asterisks indicate the first time point in which floral primordia were detected in the analysis of the corresponding genotype. Scale bar = 50 μm. (b–d) Normalized height and width of (b) Col-0, (c) *ap2-12* and (d) *rAP2-V* to the first time point of the time course shown in Fig. 1a–b. The color of the dots corresponds to the

represented parameter. The horizontal bars represent the median value for each genotype. (e) Quantification of the changes in cell area in the meristem region of Col-0, *ap2-12* and *rAP2-V* SAMs during the time course under continuous LDs. Significant differences between wild-type and mutants within each time point were determined via Mann-Whitney-Wilcoxon-test ($p < 0.05$). Significant differences among time points within each genotype were determined via Kruskal-Wallis test, followed by Dunn´s post-hoc comparisons ($p < 0.05$). White dots correspond to the median for each genotype. Data sets that share a common letter do not differ significantly. See Supplementary Data 7 for precise sample size and p-values of Mann-Whitney-Wilcoxon and Kruskal-Wallis test. Source Data are provided as a Source Data file.

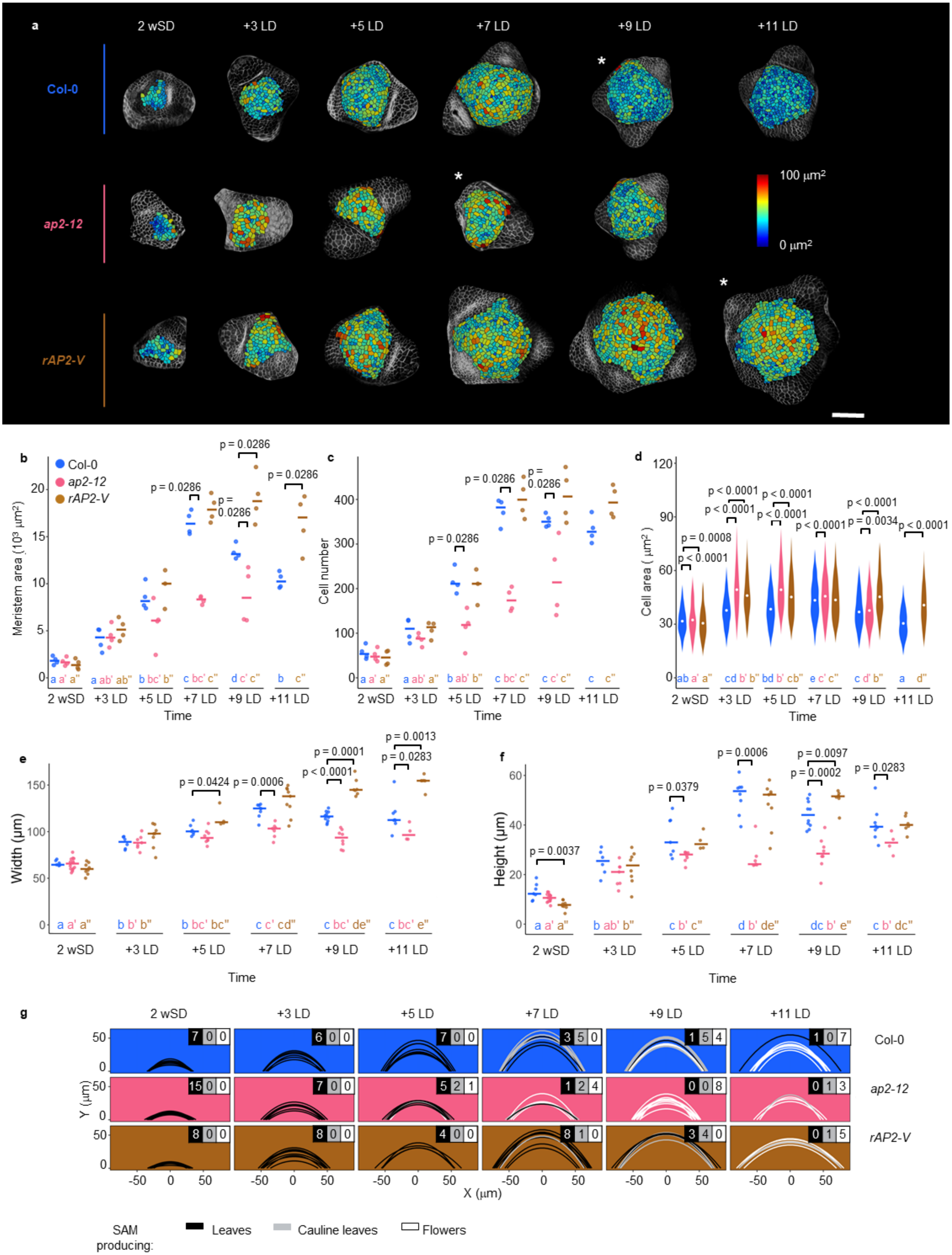
a
2 wSD
+3 LD
+5 LD
+7 LD
+9 LD
+11 LD
Col-0
ap2-12
rAP2-V
100 µm²
0 µm²
b
Col-0
ap2-12
rAP2-V
Meristem area (10³ µm²)
p = 0.0286
Time
c
Cell number
d
Cell area (µm²)
p < 0.0001
p = 0.0008
p = 0.0034
e
Width (µm)
p = 0.0424
p = 0.0006
p = 0.0001
p = 0.0013
p = 0.0283
f
Height (µm)
p = 0.0037
p = 0.0379
p = 0.0097
p = 0.0002
g
Y (µm)
X (µm)
SAM producing:
Leaves
Cauline leaves
Flowers

Supplementary Figure 2. Characterization of meristem morphology of plants grown 2 weeks under SDs and then moved under LDs to induce floral transition. (a–d) Segmentation analyses of Col-0, *ap2-12* and *rAP2-VENUS* SAMs from 2-week-old plants grown under short day (SD) conditions (2 wSD) and then transferred to long days (LD). (a) Top view of the heatmap quantification of cell area in the meristem region. White asterisks indicate the first time point at which floral primordia were detected in the analysis of the corresponding genotype. Scale bar = 50 μm. (b–c) Quantification of (b) meristem area, (c) cell number and (d) cell area. The horizontal bars represent the median value for each genotype. Significant differences between wild-type and mutants within each time point were determined via Mann-Whitney-Wilcoxon-test ($p < 0.05$). Significant differences among time points within each genotype were determined for (b) and (c) via one-way ANOVA (two-sided), followed by Tukey post-hoc comparisons ($p < 0.05$) and for (d) via Kruskal-Wallis test, followed by Dunn´s post-hoc comparisons ($p < 0.05$). Data sets that share a common letter do not differ significantly. The color of the dots and the letters correspond to the genotype. n = 4 (except *rAP2-VENUS* at +5 LD, n = 3). (e–f) Measurement of (e) width and (f) height of the SAM in 2wSD-grown plants that were then transferred to LDs. The horizontal bars represent the median value for each genotype. Significant differences between wild-type and mutants within each time point were determined via Mann-Whitney-Wilcoxon-test ($p < 0.05$). Significant differences among time points within each genotype were determined via one-way ANOVA (two-sided), followed by Tukey post-hoc comparisons ($p < 0.05$). Data sets that share a common letter do not differ significantly. The color of the dots and the letters correspond to the genotype. (g) SAM morphology adjusted to parabolas. The parabolas are colored according to the identity of primordia that were formed at the SAM periphery. The number of meristems producing each kind of primordia are listed on the top-right corner in each genotype and time point. Segmentation analyses were not performed in *ap2-12* mutants at +11 LD because they were well advanced in inflorescence development. See Supplementary Data 7 for precise sample size and p-values of Mann-Whitney-Wilcoxon, ANOVA and Kruskal-Wallis test. Source Data are provided as a Source Data file.

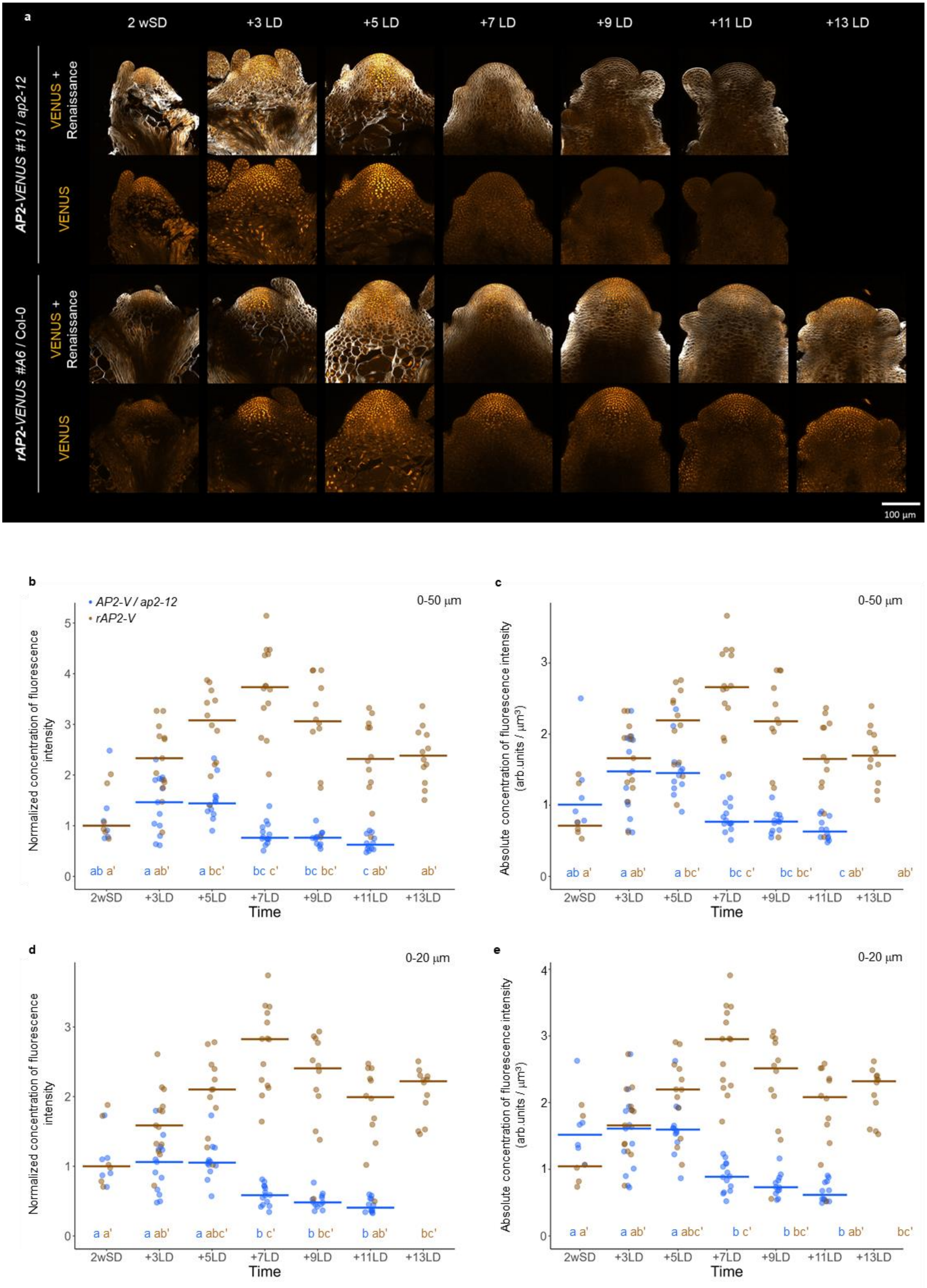
a
2 wSD
+3 LD
+5 LD
+7 LD
+9 LD
+11 LD
+13 LD
AP2-VENUS #13 / ap2-12
VENUS + Renaissance
VENUS
rAP2-VENUS #A6 / Col-0
VENUS + Renaissance
VENUS
100 µm
b
AP2-V / ap2-12
rAP2-V
0-50 µm
Normalized concentration of fluorescence intensity
ab a'
a ab'
a bc'
bc c'
bc bc'
c ab'
ab'
2wSD
+3LD
+5LD
+7LD
+9LD
+11LD
+13LD
Time
c
0-50 µm
Absolute concentration of fluorescence intensity (arb.units / µm³)
ab a'
a ab'
a bc'
bc c'
bc bc'
c ab'
ab'
Time
d
0-20 µm
Normalized concentration of fluorescence intensity
a a'
a ab'
a abc'
b c'
b bc'
b ab'
bc'
Time
e
0-20 µm
Absolute concentration of fluorescence intensity (arb.units / µm³)
a a'
a ab'
a abc'
b c'
b bc'
b ab'
bc'
Time

Supplementary Figure 3. Quantification of AP2:VENUS concentration of fluorescence intensity at the SAM of 2 wSD-grown plants transferred to LD conditions. (a) Pattern of protein accumulation of *AP2::AP2:VENUS* in *ap2-12* and *AP2::rAP2:VENUS* in Col-0 at the SAM of 2 wSD-grown plants and then transferred to continuous LDs. Scale bar = 100 μm. Either the merged VENUS and Renaissance channels or the VENUS channel are displayed. (b–e) Quantification of AP2:VENUS and rAP2:VENUS concentration of fluorescence intensity (total fluorescence divided by volume) at the shoot apex from the tip to (b–c) 50 μm or (d–e) 20 μm. The plots contain either (b, d) normalized values to the first time point or (c, e) absolute values. The dots are colored according to the mutant background of the analyzed plant. The horizontal bars represent the median value for each genotype. Significant differences among time points within each genotype were determined via one-way ANOVA (two-sided), followed by Tukey post-hoc comparisons ($p < 0.05$). Data sets that share a common letter do not differ significantly. The color of the dots and the letters correspond to the genotype. See Supplementary Data 7 for precise sample size and p-values of ANOVA test. Source Data are provided as a Source Data file.

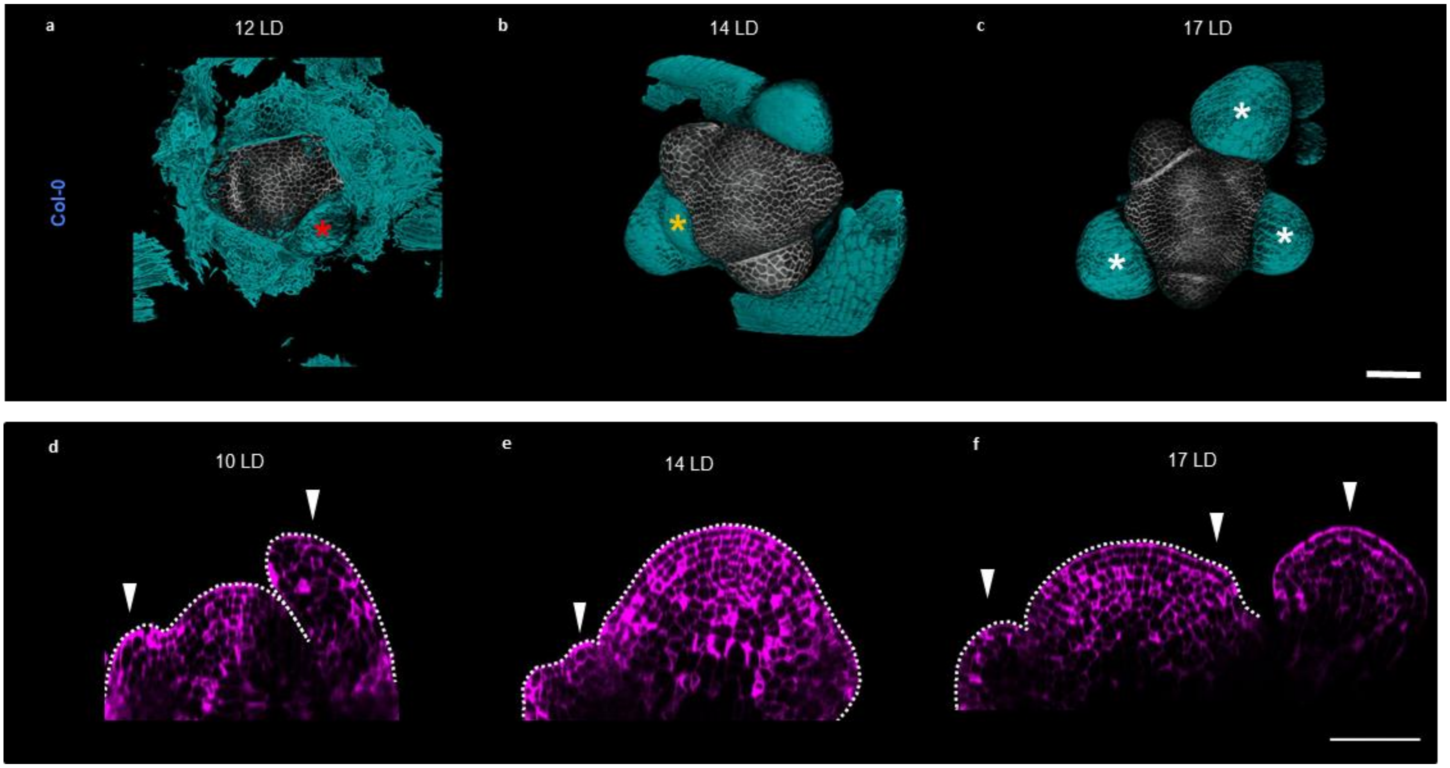

Supplementary Figure 4. Classification of the primordia formed at SAM flanks before, during and after floral transition. (a–c) Top views of the Col-0 SAM exhibiting (a) vegetative leaves (red asterisk), (b) cauline leaves (yellow asterisk) and (c) flowers (white asterisk). (d–f) Side views of the Col-0 meristem with (d) vegetative leaves, (e) cauline leaves and (f) flowers. The aforementioned primordia are indicated with white arrowheads.

The age of the SAMs is indicated on top of each image. A SAM was labeled as forming cauline leaves when a primordium corresponding to an axillary meristem with its subtending cauline leaf was unambiguously identified at the periphery of the meristem, although the younger primordia would likely be developing flowers. Scale bar = 50 μm.

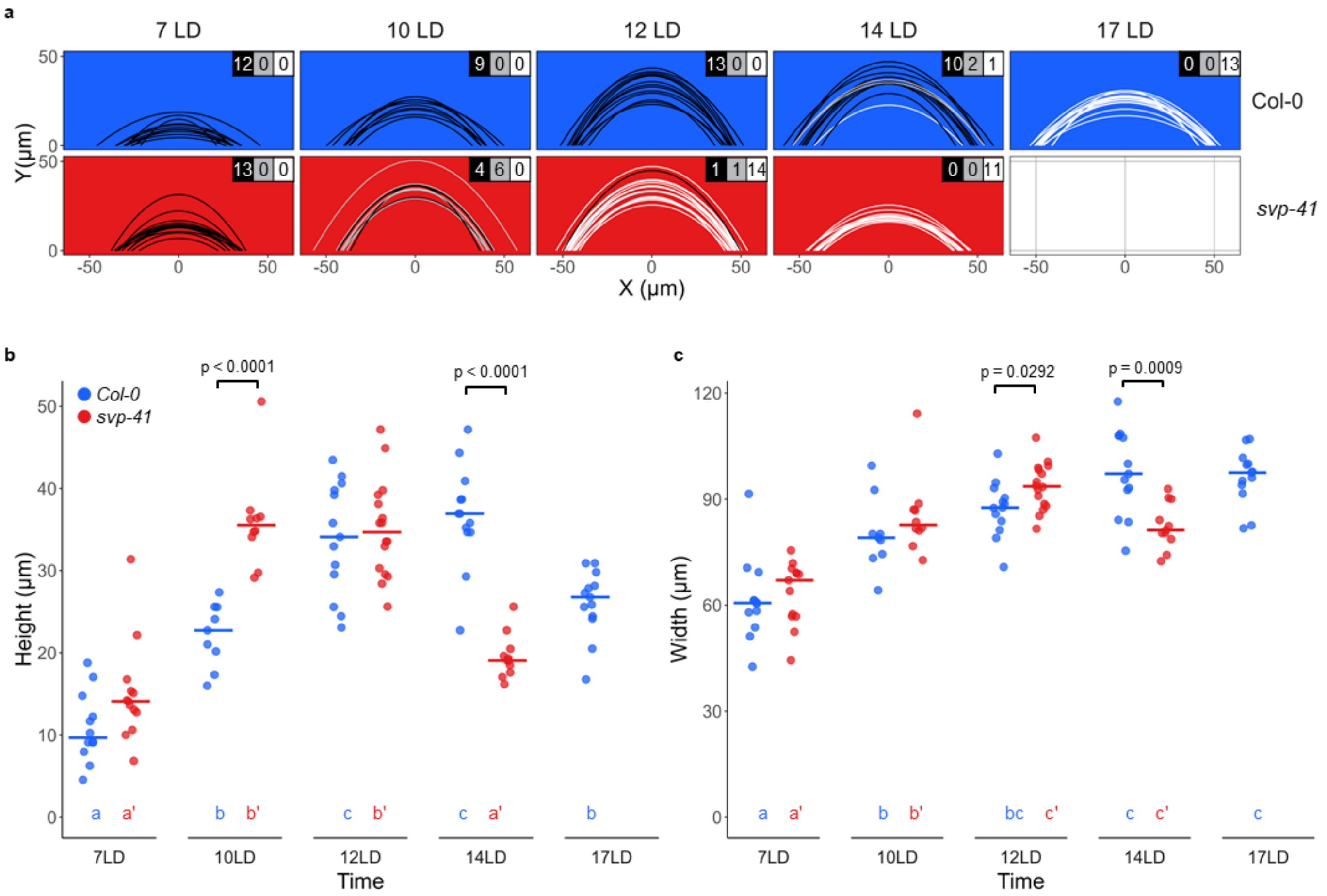

Supplementary Figure 5. Morphological changes of the SAM of *svp-41* mutants during floral transition. (a) SAM morphology adjusted to parabolas of Col-0 and *svp-41*. The parabolas are colored according to the identity of primordia that were formed at the SAM periphery. The number of meristems producing each kind of primordia are listed on the top-right corner in each genotype and time point. (b–c) Measurement of (b) height and (c) width of the SAM of Col-0 and *svp-41* plants grown under LDs. The horizontal bars represent the median value for each genotype. Significant differences between wild-type and mutants within each time point were determined via Mann-Whitney-Wilcoxon-test ($p < 0.05$). Significant differences among time points within each genotype were determined via one-way ANOVA (two-sided), followed by Tukey post-hoc comparisons ($p < 0.05$). Data sets that share a common letter do not differ significantly. The color of the dots and the letters correspond to the genotype.

See Supplementary Data 7 for precise sample size and p-values of Mann-Whitney-Wilcoxon and ANOVA test.

Source Data are provided as a Source Data file.

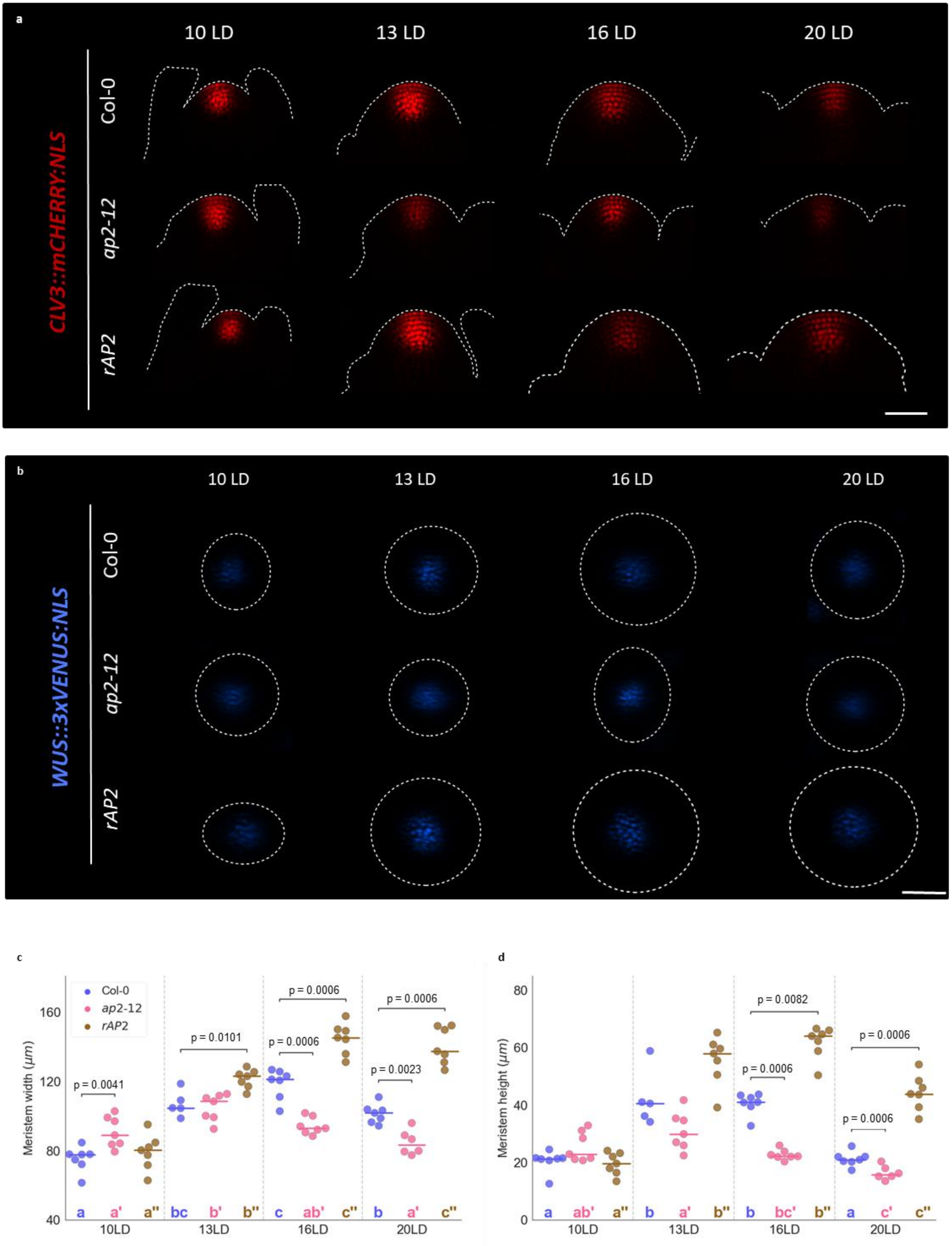
a
10 LD
13 LD
16 LD
20 LD
CLV3::mCHERRY:NLS
Col-0
ap2-12
rAP2
b
10 LD
13 LD
16 LD
20 LD
WUS::3xVENUS:NLS
Col-0
ap2-12
rAP2
c
Col-0
ap2-12
rAP2
Meristem width (μm)
p = 0.0041
p = 0.0101
p = 0.0006
p = 0.0006
p = 0.0006
p = 0.0023
a a' a'' bc b' b'' c ab' c'' b a' c''
10LD
13LD
16LD
20LD
d
Meristem height (μm)
p = 0.0082
p = 0.0006
p = 0.0006
p = 0.0006
a ab' a'' b a' b'' b bc' b'' a c' c''
10LD
13LD
16LD
20LD

Supplementary Figure 6. Supplementary data for Fig. 3. (a–b) Pattern expression of (a) *CLV3::mCHERRY:NLS* and (b) *WUS::3xVENUS-NLS* at Col-0, *ap2-12* and *rAP2* SAMs of plants grown under continuous longs days (LDs). In the (a) side views, the shape of the acquired meristem and its peripheral organs is indicated with a dotted white line, whereas in the (b) top views, the images were generated via an orthogonal projection of the xz plane for 50 μm from the top and the meristematic region was highlighted using a dotted line. Scale bar = 50 μm. (c–d) Quantification of (c) width and (d) height of the SAMs from Fig. 3. The horizontal bars represent the median value for each genotype. Significant differences between wild-type and mutants within each time point were determined via Mann-Whitney-Wilcoxon-test ($p < 0.05$). Significant differences among time points within each genotype were determined via one-way ANOVA (two-sided), followed by Tukey post-hoc comparisons ($p < 0.05$). Data sets that share a common letter do not differ significantly. The color of the dots and the letters correspond to the genotype. See Supplementary Data 7 for precise sample size and p-values of Mann-Whitney-Wilcoxon and ANOVA test. Source Data are provided as a Source Data file.

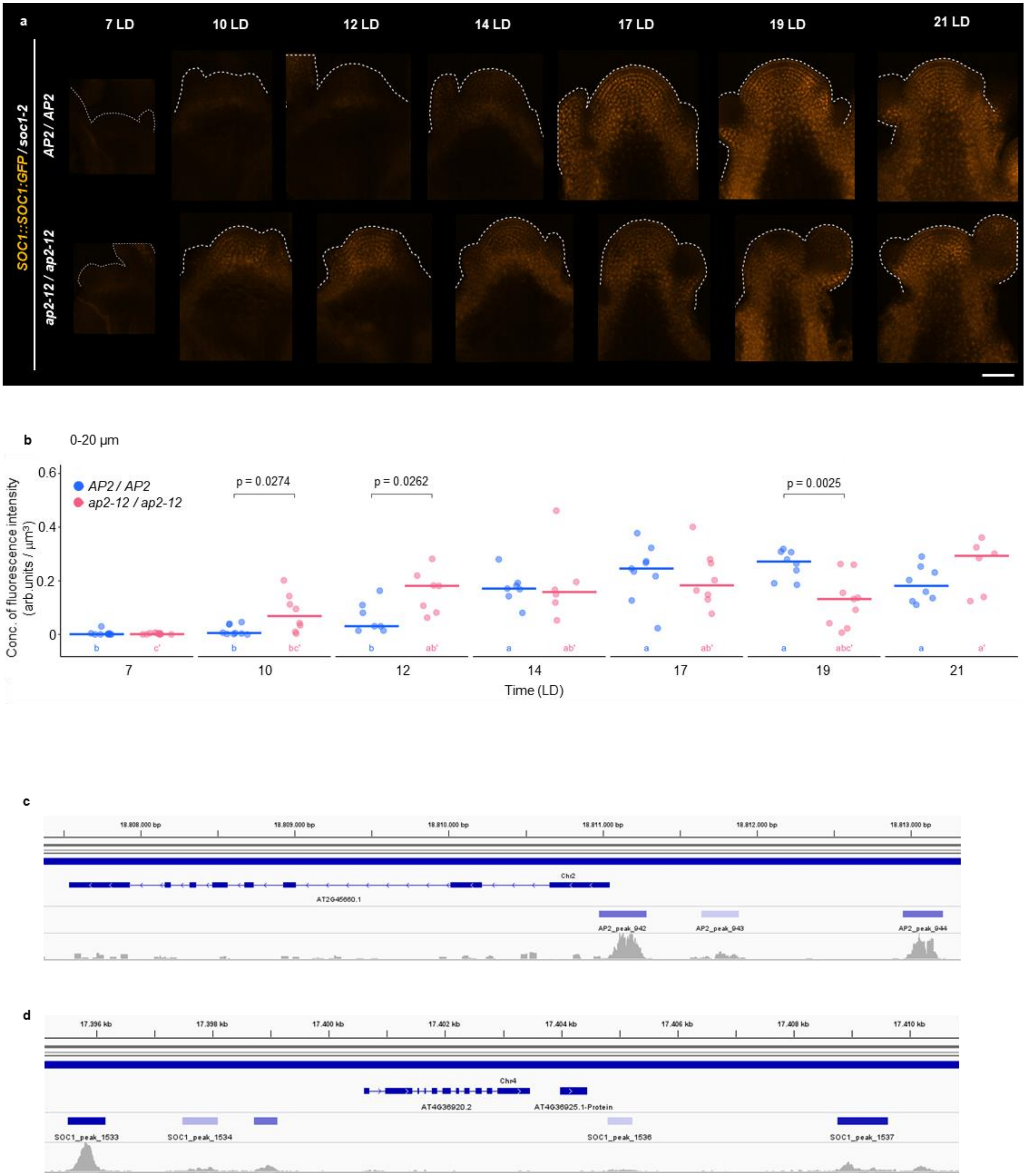

Supplementary Figure 7. Supplementary data to Fig. 4. (a) Images without clipping depicting the pattern of protein accumulation of *SOC1::SOC1:GFP* in *soc1-2* and *soc1-2 ap2-12* mutant backgrounds at the SAM of plants grown under continuous long day (LD) conditions, shown in Fig. 3c. The shape of the acquired meristem and its peripheral organs is indicated with a dotted white line. Scale bar = 50 μm. (b) Quantification of SOC1:GFP

normalized fluorescence at the shoot apex (from the tip to 20 μm deep in the basal direction) in *soc1-2* and *soc1-2 ap2-12* mutant backgrounds during continuous LDs. The dots are colored according to the mutant background of the analyzed plant. Significant differences between wild-type and mutants within each time point were determined via Mann-Whitney-Wilcoxon-test ($p < 0.05$). Significant differences among time points within each genotype were determined via one-way ANOVA (two-sided), followed by Tukey post-hoc comparisons ($p < 0.05$).See Supplementary Data 7 for precise sample size and p-values of Mann-Whitney-Wilcoxon and ANOVA test. Source Data are provided as a Source Data file. (c-d) GBrowse traces of mapped ChIP-Seq reads for AP2 and SOC1. Previously published ChIP-seq data for AP2[36] and SOC1[20] were reanalyzed. Binding of (c) AP2 to the promoter region of *SOC1* and (d) SOC1 to the promoter region of *AP2*. Gene models are shown under the upper scale bar. The blue horizontal bars below the track of the gene models delimit the position of the peak regions. The coverage of the mapped reads is represented in the lower tracks in each subfigure by gray bars.

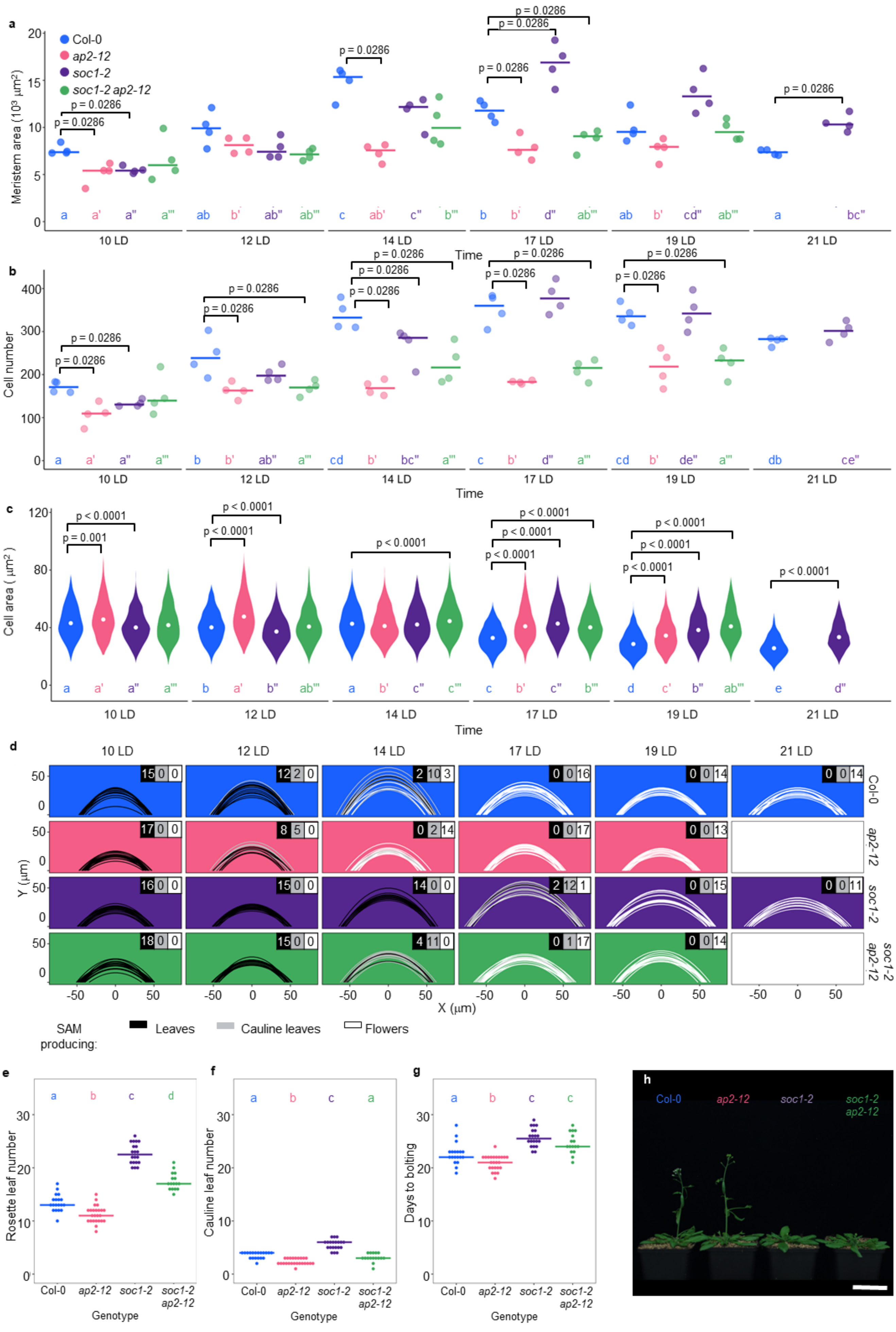
a
Col-0
ap2-12
soc1-2
soc1-2 ap2-12
Meristem area (10³ µm²)
p = 0.0286
10 LD
12 LD
14 LD
17 LD
19 LD
21 LD
Time
b
Cell number
c
Cell area (µm²)
p < 0.0001
p = 0.001
d
Y (µm)
X (µm)
SAM producing:
Leaves
Cauline leaves
Flowers
e
Rosette leaf number
Genotype
f
Cauline leaf number
g
Days to bolting
h

Supplementary Figure 8. Supplementary data to Fig. 5: morphological analyses and flowering time of *soc1-2 ap2-12.* (a–c) Quantification of (a) meristem area, (b) cell number and (c) cell area in the meristematic region shown in Fig. 4a. The horizontal bars (a–b) and white dots (c) represent the median value for each genotype. Significant differences between wild-type and mutants within each time point were determined via Mann-Whitney-Wilcoxon-test ($p < 0.05$). Significant differences among time points within each genotype were determined for (a) and (b) via one-way ANOVA (two-sided), followed by Tukey post-hoc comparisons ($p < 0.05$) and for (c) via Kruskal-Wallis test, followed by Dunn´s post-hoc comparisons ($p < 0.05$). Data sets that share a common letter do not differ significantly. The color of the dots, the shapes denoting the data and the letters correspond to the genotype. (d) SAM morphology of Col-0, *ap2-12*, *soc1-2* and *soc1-2 ap2-12* under continuous LDs. The parabolas are colored according to the identity of primordia that were formed at the SAM periphery. The number of meristems producing each kind of primordia are listed on the top-right corner in each genotype and time point. (e–h) Flowering-time analysis of Col-0, *ap2-12*, *soc1-2* and *soc1-2 ap2-12* under long days (LDs). (e–g) Flowering time according to (e) rosette leaf number, (f) cauline leaf number and (g) days to bolting. The horizontal bars represent the median value for each genotype. Significant differences among genotypes were determined via one-way ANOVA (two-sided), followed by Tukey post-hoc comparisons ($p < 0.05$). Data sets that share a common letter do not differ significantly. (h) Photograph of representative plants of the used genotypes grown for 27 days under LD conditions. Scale bar = 5 cm. See Supplementary Data 7 for precise sample size and p-values of Mann-Whitney-Wilcoxon, ANOVA and Kruskal-Wallis test. Source Data are provided as a Source Data file.

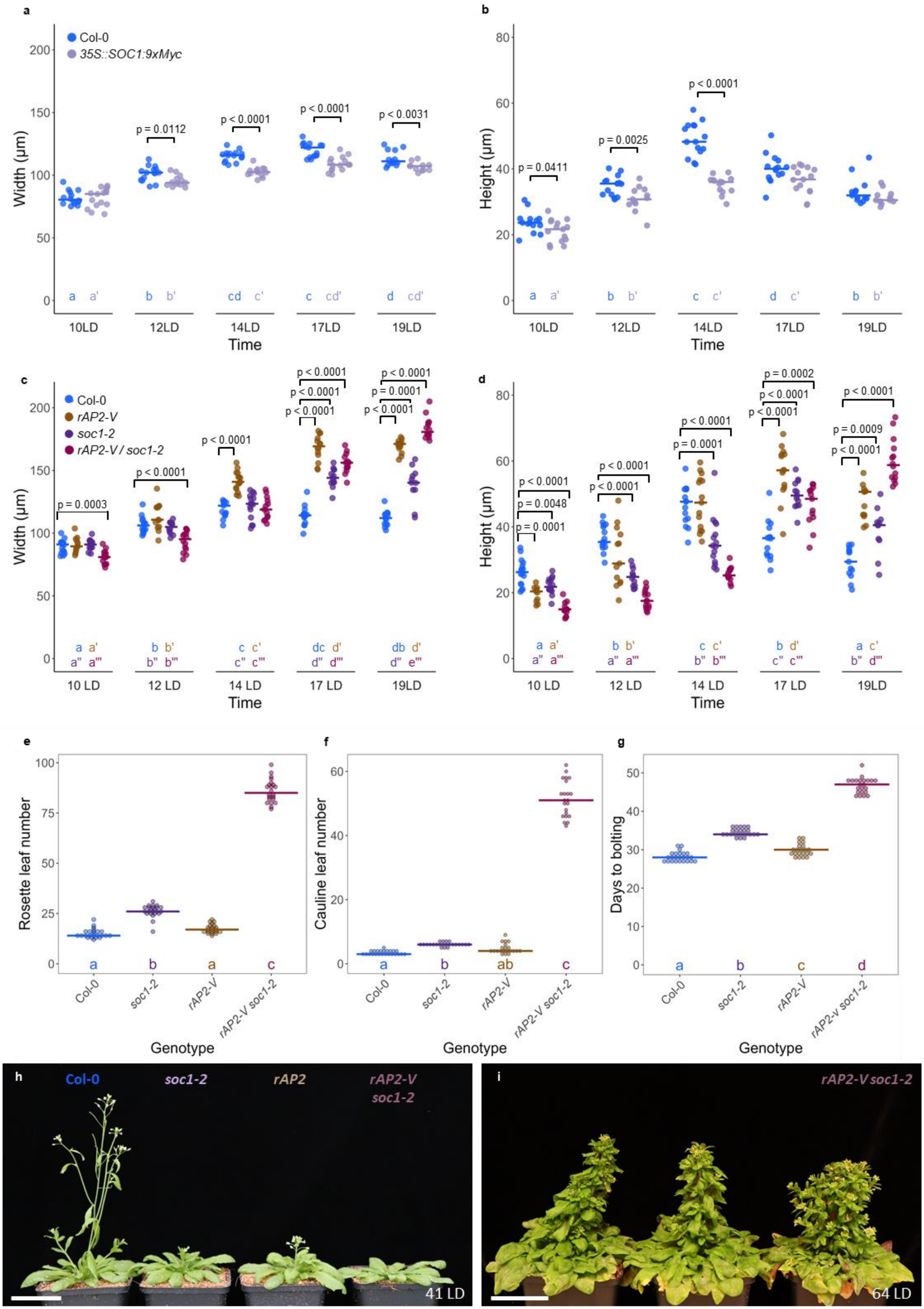

a
Col-0
35S::SOC1:9xMyc
Width (µm)
p = 0.0112
p < 0.0001
p < 0.0001
p < 0.0031
a a'
b b'
cd c'
c cd'
d cd'
10LD
12LD
14LD
17LD
19LD
Time
b
Height (µm)
p = 0.0411
p = 0.0025
p < 0.0001
a a'
b b'
c c'
d c'
b b'
10LD
12LD
14LD
17LD
19LD
Time
c
Col-0
rAP2-V
soc1-2
rAP2-V / soc1-2
Width (µm)
p = 0.0003
p < 0.0001
p < 0.0001
p < 0.0001
p < 0.0001
p < 0.0001
p < 0.0001
p = 0.0001
p < 0.0001
a a' a'' a'''
b b' b'' b'''
c c' c'' c'''
dc d' d'' d'''
db d' d'' e'''
10 LD
12 LD
14 LD
17 LD
19LD
Time
d
Height (µm)
p < 0.0001
p = 0.0048
p = 0.0001
p < 0.0001
p < 0.0001
p < 0.0001
p = 0.0001
p = 0.0002
p < 0.0001
p < 0.0001
p < 0.0001
p = 0.0009
p < 0.0001
a a' a'' a'''
b b' a'' a'''
c c' b'' b'''
b d' c'' c'''
a c' b'' d'''
10 LD
12 LD
14 LD
17 LD
19LD
Time
e
Rosette leaf number
a b a c
Col-0
soc1-2
rAP2-V
rAP2-V soc1-2
Genotype
f
Cauline leaf number
a b ab c
Col-0
soc1-2
rAP2-V
rAP2-V soc1-2
Genotype
g
Days to bolting
a b c d
Col-0
soc1-2
rAP2-V
rAP2-v soc1-2
Genotype
h
Col-0
soc1-2
rAP2
rAP2-V soc1-2
41 LD
i
rAP2-V soc1-2
64 LD

Supplementary Figure 9. Supplementary data to Fig. 5: morphological analysis of *35S::SOC1:9xMYC* and *rAP2-V soc1-2*, and flowering time of *rAP2-V soc1-2.* (a–d) Quantification of (a, d) width and (b, d) height of (a–b) *35S::SOC1:9xMYC* and (c–d) *rAP2-V soc1-2.* Significant differences between wild-type and mutants within each time point were determined via Mann-Whitney-Wilcoxon-test ($p < 0.05$). Significant differences among time points within each genotype were determined via one-way ANOVA (two-sided), followed by Tukey post-hoc comparisons ($p < 0.05$). Data sets that share a common letter do not differ significantly. The color of the dots and the letters correspond to the genotype. (e–i) Flowering time of *rAP2-V soc1-2* according to (e) rosette leaf number, (f) cauline leaf number and (g) days to bolting. The horizontal bars represent the median value for each genotype. Significant differences among genotypes were determined via one-way ANOVA (two-sided), followed by Tukey post-hoc comparisons ($p < 0.05$). Data sets that share a common letter do not differ significantly. (h–i) Photograph of representative plants of the used genotypes grown for (h) 41 and (i) 64 days under LD conditions. Scale bar = 5 cm. See Supplementary Data 7 for precise sample size and p-values of Mann-Whitney-Wilcoxon and ANOVA test. Source Data are provided as a Source Data file.

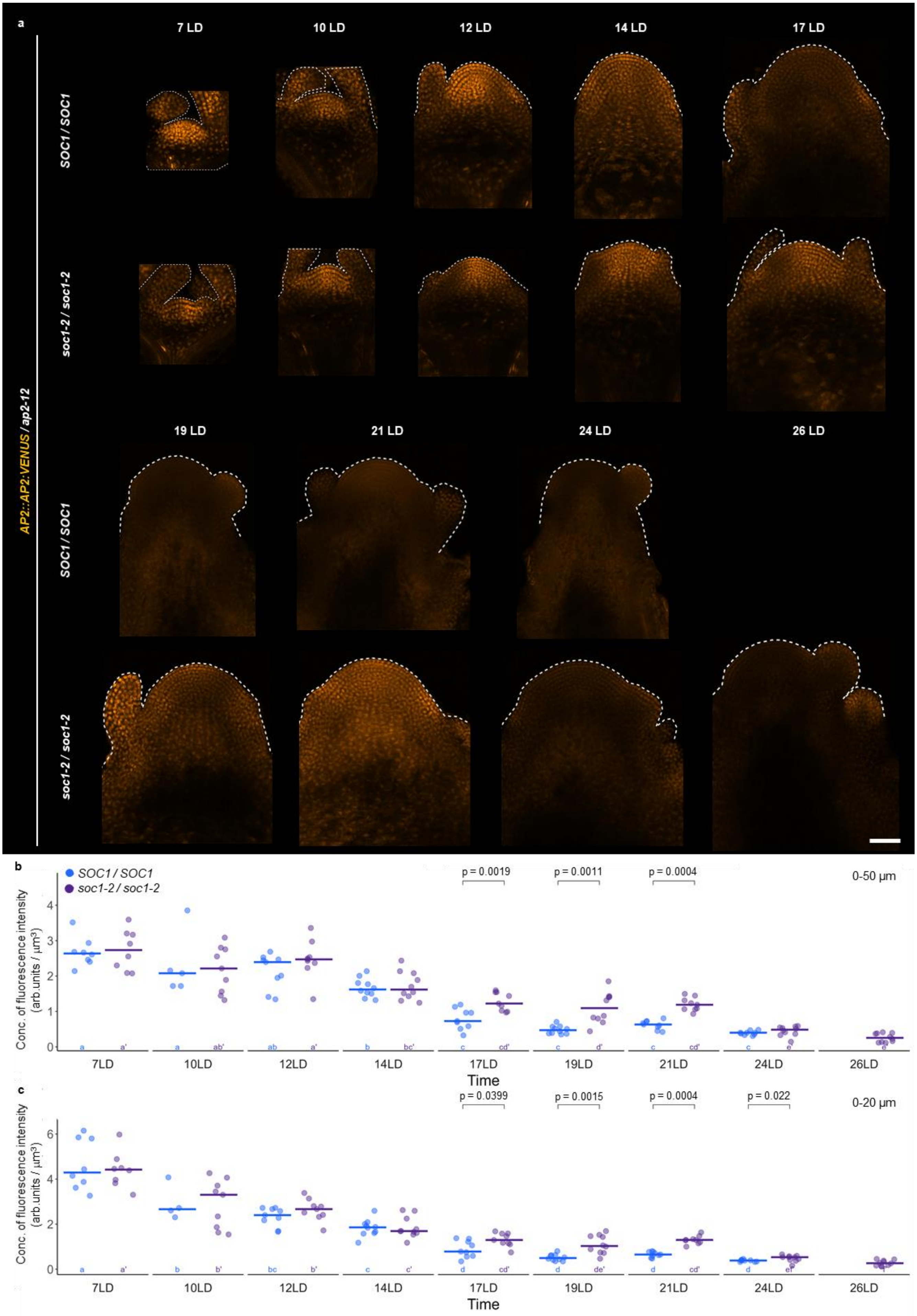

a
7 LD
10 LD
12 LD
14 LD
17 LD
19 LD
21 LD
24 LD
26 LD
SOC1 / SOC1
soc1-2 / soc1-2
AP2::AP2:VENUS / ap2-12
b
SOC1 / SOC1
soc1-2 / soc1-2
0-50 µm
p = 0.0019
p = 0.0011
p = 0.0004
Conc. of fluorescence intensity (arb.units / µm³)
7LD
10LD
12LD
14LD
17LD
19LD
21LD
24LD
26LD
Time
c
0-20 µm
p = 0.0399
p = 0.0015
p = 0.0004
p = 0.022
Conc. of fluorescence intensity (arb.units / µm³)
Time

Supplementary Figure 10. Supplementary data to Fig. 6. (a) Images without clipping depicting the pattern of protein accumulation of *AP2::AP2:VENUS* in *ap2-12* and *ap2-12 soc1-2* mutants at the SAM of plants grown under continuous long day (LD) conditions shown in Fig. 6a. The shape of the acquired meristem and its peripheral organs is indicated with a dotted white line. Scale bar = 50 μm. (b) Quantification of AP2:VENUS normalized fluorescence at the shoot apex from the tip to (b) 50 μm (shown also in Fig. 6b with the extra time point of 26 LD) and (c) 20 μm deep in the basal direction in *ap2-12* and *soc1-2 ap2-12* mutants during continuous LDs. The dots are colored according to the mutant background of the analyzed plant. Significant differences between wild-type and mutants within each time point were determined via Mann-Whitney-Wilcoxon-test (p < 0.05). Significant differences among time points within each genotype were determined via one-way ANOVA (two-sided), followed by Tukey post-hoc comparisons (p < 0.05). Data sets that share a common letter do not differ significantly. The color of the dots and the letters correspond to the genotype. See Supplementary Data 7 for precise sample size and p-values of Mann-Whitney-Wilcoxon and ANOVA test. Source Data are provided as a Source Data file.

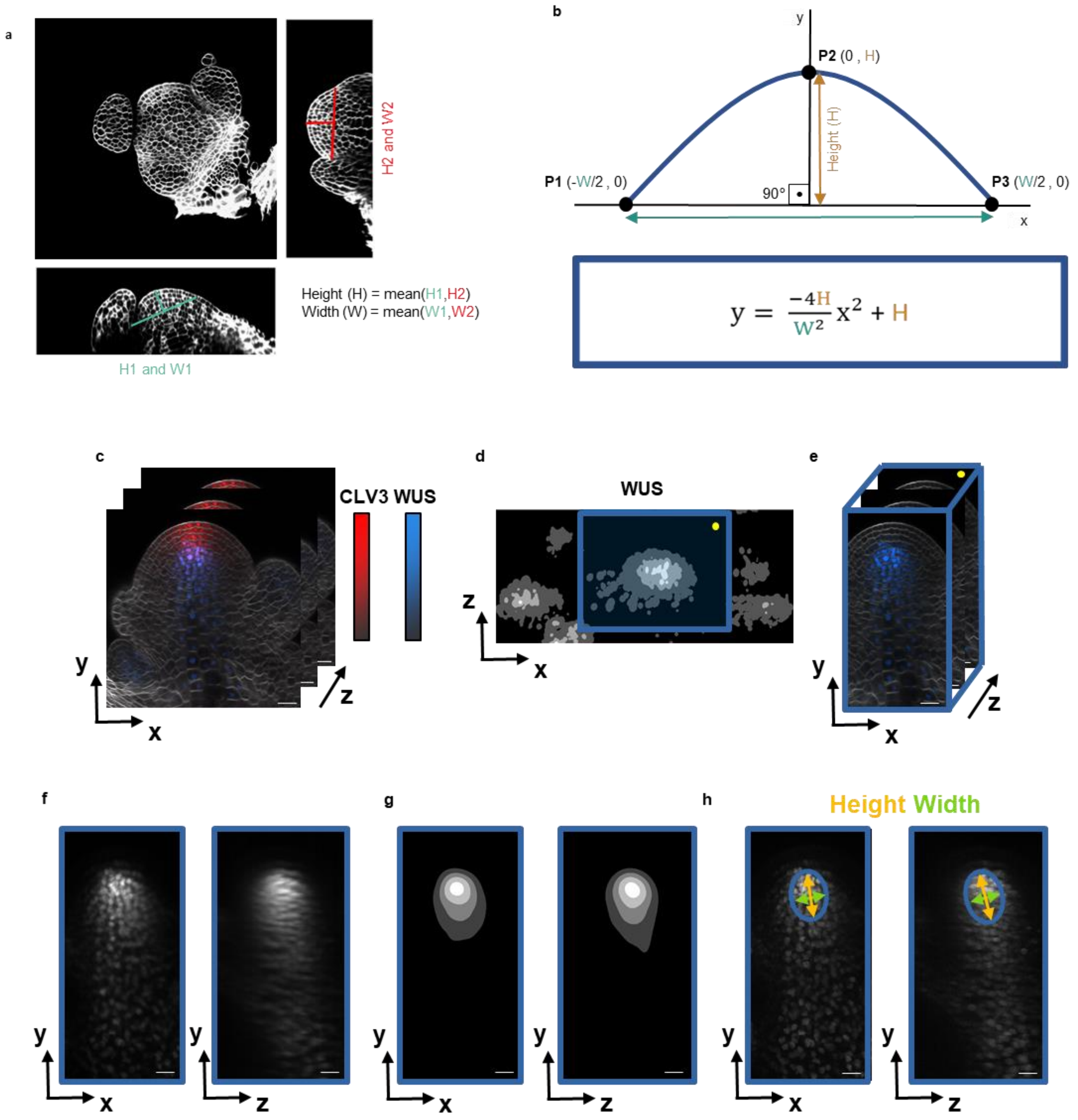

Supplementary Figure 11. Methodology for the morphological characterization of the meristem morphology and the *WUS* and *CLV3* domains of expression. (a–b) Pipeline for the SAM morphological quantification. (a) Two orthogonal views were generated from each z-stack. In each orthogonal view, SAM height and width were measured following the specified criteria in materials and methods. The two measurements for each parameter were considered as technical replicates; therefore, the mean of these values was used for further analysis. (b) Adjustment of the measured height and width to a parabola, (b, top) represented in a cartesian coordinate

system. Notice that three points can be placed at the extremes of these measurements, which define a unique parabola. (b, bottom) Equation to represent a parabola from the measured height and width. (c–h) Pipeline for the quantification of the *WUS* and *CLV3* domains of expression within the meristem. (c) The pipeline uses SAM fluorescence confocal microscopy images with both *CLV3::mCHERRY:NLS* and *WUS::3xVENUS:NLS*. For the presented example, we consider the *WUS* expression of a SAM harvested at 16 long days (LD). (d) This panel shows a screenshot of a top (xz) view a of the z-stack projection; the blue line shows the smaller region of interest that is selected to exclude signal from new primordia. (e) New stack produced which does not include the primordia signal. (f) Orthogonal SUM projections of the signal contained in the reduced stack. (g) Isocline representation to show the different levels of transcription in the expression domain. The colormap goes from the region with the highest 10% expression levels in white, followed by gradually darker regions delimiting the 30%, 50% and the 70% fluorescence levels. (h) Graphical representation of the ellipses fitted to the >50% level region with two colored arrows showing the height and width of the domain for the two orthogonal projections.

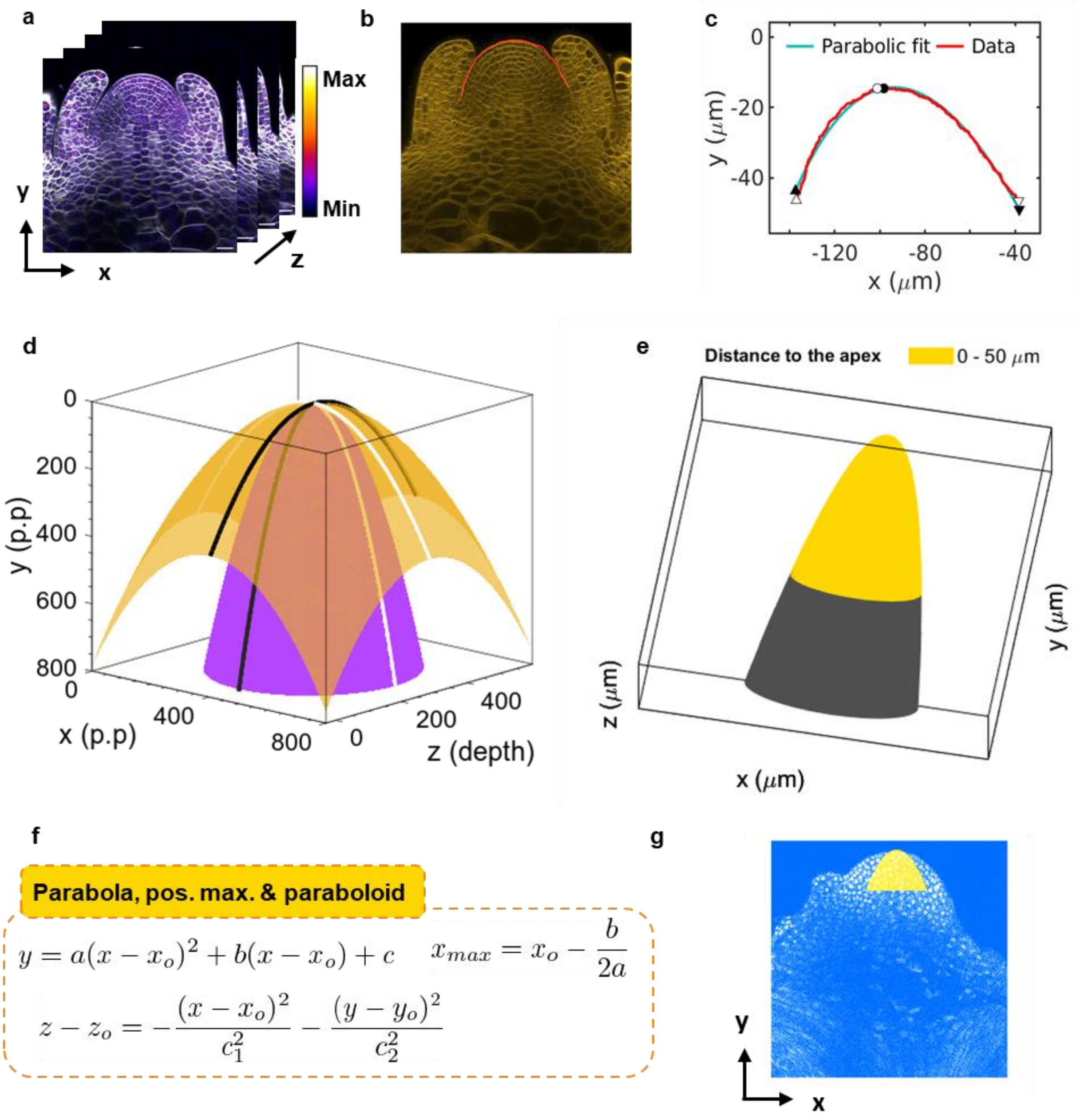

Supplementary Figure 12. Methodology to quantify mean fluorescence intensity values of *AP2-VENUS* and *SOC1-GFP* within the SAM. (a) SAM fluorescence confocal microscopy images were obtained from the lateral side. In the image: slices from a z-stack meristem from a 12-day-old plant grown under long day conditions (LDs) containing the *AP2::AP2:VENUS* reporter. The membrane marker channel is displayed in white and the AP2-VENUS fluorescence signal is colored according to the Fire color look-up table in Fiji. Scale bar = 20 μm. The acquisition parameters are described in Materials and Methods. (b) A curved line (in red) marks the meristem outline until the boundaries of the primordia closest to the SAM tip on the sum of slice projection of a certain z-stack slice interval. (c) Parabolic fitting of the parabola shown in (b). Triangles mark the beginning and end points of the drawn curve line (white) and fitted parabola (black). Circles represent the apex position for the drawn curve

line (white) and fitted parabola (black). (d) 3D paraboloids built from the previously extracted orthogonal parabolas. In orange, paraboloid using the original curvature values, with the extracted orthogonal parabolas outlined on top of it; in purple, paraboloid with higher curvature to focus on the central area of the meristem along its longitudinal axis. The purple paraboloid is used to generate a mask such that when multiplied to a given z-stack, the intensity values of pixels whose positions lie outside the paraboloid are set to zero. p.p. = pixel position. (e) 3D-paraboloid and, in orange, the section of interest (0 - 50μm distance from the apex coordinates) in which the fluorescence intensity is quantified. f) Main set of equations describing the 2D parabola in the xy-plane, its maximum position and the 3D paraboloid. The 2D equation of the parabola in the zy plane is omitted for simplicity. (g) Representation of the SAM region used for the signal quantification (yellow) in Fig. 4 and Fig. 6.
