# Supplementary material for: Coordination of shoot apical meristem shape and identity by APETALA2 during floral transition in Arabidopsis
Source: Nat Commun. 2024 Aug 13;15:6930. doi: 10.1038/s41467-024-51341-6 (PMC11322546; doi:10.1038/s41467-024-51341-6)
Supplement: Supplementary file 3 — Description of additional supplementary files [file 41467_2024_51341_MOESM3_ESM.pdf]

Description of Additional Supplementary Files

Supplementary Data 1. List of differentially expressed genes in ap2-12 vs. Col-0 at least at one time point during the RNA-seq time course.

Supplementary Data 2. List of differentially expressed genes in ap2-12 vs. Col-0 at 14 LD.

Supplementary Data 3. List of differentially expressed genes in Col-0 at 14 LD vs. 10 LD.

Supplementary Data 4. List of genes that are present in Supplementary Data 2 and Supplementary Data 3.

Supplementary Data 5. List of direct target genes of AP2 identified by ChIP-Seq analysis in36 .

Supplementary Data 6. Oligonucleotides used in this study.

Supplementary Data 7. Sample size (n) and statistical significance (p-value) of the analyses of this manuscript.
