# Supplementary material for: Coordination of shoot apical meristem shape and identity by APETALA2 during floral transition in Arabidopsis
Source: Nat Commun. 2024 Aug 13;15:6930. doi: 10.1038/s41467-024-51341-6 (PMC11322546; doi:10.1038/s41467-024-51341-6)
Supplement: Supplementary file 11 — Reporting Summary [file 41467_2024_51341_MOESM11_ESM.pdf]

nature portfolio

Corresponding author(s): George Coupland

Last updated by author(s): Jul 29, 2024

Reporting Summary

Nature Portfolio wishes to improve the reproducibility of the work that we publish. This form provides structure for consistency and transparency in reporting. For further information on Nature Portfolio policies, see our Editorial Policies and the Editorial Policy Checklist.

Statistics

For all statistical analyses, confirm that the following items are present in the figure legend, table legend, main text, or Methods section.

n/a	Confirmed	
☐	☒	The exact sample size (*n*) for each experimental group/condition, given as a discrete number and unit of measurement
☐	☒	A statement on whether measurements were taken from distinct samples or whether the same sample was measured repeatedly
☐	☒	The statistical test(s) used AND whether they are one- or two-sided<br>*Only common tests should be described solely by name; describe more complex techniques in the Methods section.*
☒	☐	A description of all covariates tested
☐	☒	A description of any assumptions or corrections, such as tests of normality and adjustment for multiple comparisons
☐	☒	A full description of the statistical parameters including central tendency (e.g. means) or other basic estimates (e.g. regression coefficient) AND variation (e.g. standard deviation) or associated estimates of uncertainty (e.g. confidence intervals)
☐	☒	For null hypothesis testing, the test statistic (e.g. *F*, *t*, *r*) with confidence intervals, effect sizes, degrees of freedom and *P* value noted<br>*Give P values as exact values whenever suitable.*
☒	☐	For Bayesian analysis, information on the choice of priors and Markov chain Monte Carlo settings
☒	☐	For hierarchical and complex designs, identification of the appropriate level for tests and full reporting of outcomes
☒	☐	Estimates of effect sizes (e.g. Cohen's *d*, Pearson's *r*), indicating how they were calculated

Our web collection on statistics for biologists contains articles on many of the points above.

Software and code

Policy information about availability of computer code

Data collection	No software was used.
Data analysis	Segmentation analyses: MorphoGraphX 2.0. Morphology analyses: ImageJ 1.53v. Plots and statistical analyses: R version 4.2.1. RNA-Seq analysis: CutAdapt, Trimmomatic, Salmon, DESeq2. Fluorescence quantification: https://gitlab.com/slcu/teamHJ/pau/RegionsAnalysis

For manuscripts utilizing custom algorithms or software that are central to the research but not yet described in published literature, software must be made available to editors and reviewers. We strongly encourage code deposition in a community repository (e.g. GitHub). See the Nature Portfolio guidelines for submitting code & software for further information.

Data

Policy information about availability of data

All manuscripts must include a data availability statement. This statement should provide the following information, where applicable:

- Accession codes, unique identifiers, or web links for publicly available datasets
- A description of any restrictions on data availability
- For clinical datasets or third party data, please ensure that the statement adheres to our policy

The RNA-Seq data generated in this study have been deposited in NCBI Gene Expression Omnibus database under the accession code PRJNA954448 [link]. Raw data from RNA-seq series is available (PRJNA954448). Other raw data and the original confocal microscope images are available on Edmond database under the accession code 3.G0AEP5_2024 (link) . Source data are provided with this paper.

Research involving human participants, their data, or biological material

Policy information about studies with human participants or human data. See also policy information about sex, gender (identity/presentation), and sexual orientation and race, ethnicity and racism.

Reporting on sex and gender	This study did not involve data nor biological material from human participants.
Reporting on race, ethnicity, or other socially relevant groupings	This study did not involve data nor biological material from human participants.
Population characteristics	This study did not involve data nor biological material from human participants.
Recruitment	This study did not involve data nor biological material from human participants.
Ethics oversight	This study did not involve data nor biological material from human participants.

Note that full information on the approval of the study protocol must also be provided in the manuscript.

Field-specific reporting

Please select the one below that is the best fit for your research. If you are not sure, read the appropriate sections before making your selection.

☒ Life sciences ☐ Behavioural & social sciences ☐ Ecological, evolutionary & environmental sciences

For a reference copy of the document with all sections, see nature.com/documents/nr-reporting-summary-flat.pdf

Life sciences study design

All studies must disclose on these points even when the disclosure is negative.

Sample size	Previous studies have shown that three meristems per time point and genotype are sufficient to report changes in SAM area during floral transition. At least 15 meristems were harvested per time point and genotype in order to achieve N = 4 in MorphoGraphX analyses. The maximum number of meristems feasible was analysed for morphology analyses. For flowering time experiments, 24 plants per genotype were grown. Damaged plants during the experiment were discarded.
Data exclusions	Due to either defects in the meristem sample (i.e breakage of the tissue, leaf primordia blocking the fluorescence signal,...) and/or strong developmental differences with respect to the rest of the samples within the same time point, some meristems were not considered for fluorescence quantification. Severely damaged meristems were not considered for morphology analyses.
Replication	The experiments of this study reproduced previously published results. The references to these studies are included in the main text. The affected meristem area and morphology of the meristem of ap2 mutants has been checked in three independent experiments in this study.
Randomization	All the plants used in each experiment were grown in parallel in the same conditions. The position of the pots was randomised in the allocated growing space to avoid possible bias due to position of the pots. Among all the imaged meristems, 4 SAMs per time point and genotype were selected for MorphoGraphX analysis. The meristems were selected by absence of damage, absence of developing organs covering the meristem and overall quality of the image. When more than 4 meristems were suitable for MorphoGraphX analysis, the four first imaged meristems were arbitrarily selected.
Blinding	The used material in this study was rigorously labelled, thus making blinding not possible.

Reporting for specific materials, systems and methods

We require information from authors about some types of materials, experimental systems and methods used in many studies. Here, indicate whether each material, system or method listed is relevant to your study. If you are not sure if a list item applies to your research, read the appropriate section before selecting a response.

Materials & experimental systems

n/a	Involved in the study
☒	☐ Antibodies
☒	☐ Eukaryotic cell lines
☒	☐ Palaeontology and archaeology
☒	☐ Animals and other organisms
☒	☐ Clinical data
☒	☐ Dual use research of concern
☐	☒ Plants

Methods

n/a	Involved in the study
☒	☐ ChIP-seq
☒	☐ Flow cytometry
☒	☐ MRI-based neuroimaging

Dual use research of concern

Policy information about dual use research of concern

Hazards

Could the accidental, deliberate or reckless misuse of agents or technologies generated in the work, or the application of information presented in the manuscript, pose a threat to:

No	Yes
☒	☐ Public health
☒	☐ National security
☒	☐ Crops and/or livestock
☒	☐ Ecosystems
☒	☐ Any other significant area

Experiments of concern

Does the work involve any of these experiments of concern:

No	Yes
☒	☐ Demonstrate how to render a vaccine ineffective
☒	☐ Confer resistance to therapeutically useful antibiotics or antiviral agents
☒	☐ Enhance the virulence of a pathogen or render a nonpathogen virulent
☒	☐ Increase transmissibility of a pathogen
☒	☐ Alter the host range of a pathogen
☒	☐ Enable evasion of diagnostic/detection modalities
☒	☐ Enable the weaponization of a biological agent or toxin
☒	☐ Any other potentially harmful combination of experiments and agents

Plants

Seed stocks	Most of the material that was used in this study was previously published by other research groups [ap2-12 (Yant et al. 2010), soc1-2 (Lee et al. 2000) and SOC1::SOC1:GFP / soc1-2 (Immink et al. 2012)] or by our group [AP2::AP2:9A:V #13 / ap2-12 (Ó'Maoiléidigh et al. 2021); AP2::rAP2:9A:V #A6 / Col-0 and AP2::rAP2 B2 / Col-0 (Sang, Vayssières, et al. 2022); CLV3::mCHERRY-NLS WUS::3xVENUS-NLS (Pfeiffer et al. 2016)]
Novel plant genotypes	Novel plant genotypes soc1-2 ap2-12, SOC1::SOC1:GFP / soc1-2 ap2-12 and AP2::AP2:9A:VENUS / ap2-12, CLV3::mCHERRY-NLS WUS::3xVENUS-NLS/ ap2-12 and CLV3::mCHERRY-NLS WUS::3xVENUS-NLS / rAP2 B2 were generated by crossing.
Authentication	All the used genetic material was genotyped by PCR.
